# Association between android fat mass, gynoid fat mass and cardiovascular and all-cause mortality in adults: NHANES 2003–2007

**DOI:** 10.3389/fcvm.2023.1055223

**Published:** 2023-05-18

**Authors:** Wenzhi Ma, Huiping Zhu, Xinyi Yu, Xiaobing Zhai, Shiyang Li, Nian Huang, Keyang Liu, Kokoro Shirai, Haytham A. Sheerah, Jinhong Cao

**Affiliations:** ^1^ School of Public Health, Wuhan University, Wuhan, China; ^2^ School of Public Health, Capital Medical University, Beijing, China; ^3^ Center for Artificial Intelligence Driven Drug Discovery, Faculty of Applied Sciences, Macao Polytechnic University, Macao, China; ^4^ Public Health, Department of Social Medicine, Osaka University Graduate School of Medicine, Suita-shi, Japan; ^5^ Assistant Deputyship for International Collaborations, Ministry of Health, Riyadh, Saudi Arabia; ^6^ School of Management, Hubei University of Chinese Medicine, Wuhan, China; ^7^ Research Center for the Development of Chinese Medicine, Hubei Province Project of Key Research Institute of Humanities and Social Sciences at Universities, Wuhan, China

**Keywords:** android fat mass, gynoid fat mass, cardiovascular diseases, all-cause mortality, NHANES

## Abstract

**Objectives:**

*Evidence of the relationship* between android fat mass and gynoid fat mass with the mortality prediction is still limited. Current study analyzed the NHANES database to investigate the relationship between android fat mass, gynoid fat mass and CVD, with all-cause mortality.

**Method:**

The study subjects were NHANES participants over 20 years old, two indicators of regional body composition, android fat and gynoid fat were measured by Dual Energy x-ray Absorptiometry (DEXA). The other various covariates data obtained from the NHANES questionnaire and laboratory measurements, including age, gender, education, race/ethnicity, uric acid, total serum cholesterol, albumin, Vitamin C, folate, alcohol drinking, smoking status, history of diabetes, and hypertension. Mortality status was ascertained from a linked mortality file prepared by the National Center for Health Statistics. The study population was divided quartiles based on the distribution of android fat mass and gynoid fat mass. The relationship between these two indicators with cardiovascular and all-cause mortality was investigated by using Cox regression. The covariates age, gender, smoking status, drinking status, history of diabetes, and history of hypertension were stratified.

**Results:**

In the fully adjusted model, Q3 had the lowest HR in android fat mass and gynoid fat mass. When examining the relationship between android fat mass and CVD mortality, current smokers and drinkers had the lowest CVD risk in Q2 [smoking: 0.21 (0.08, 0.52), drinking: 0.14 (0.04, 0.50)]. In diabetic patients, compared with Q1, other groups with increased android fat mass can significantly reduce the risk of CVD [Q4: 0.17 (0.04, 0.75), Q3: 0.18 (0.03, 1.09), Q2: 0.27 (0.09, 0.83)]. In ≥60 years old and female, the greater the gynoid fat mass, the smaller the HR of all-cause mortality [Q4 for ≥60 years old: 0.57 (0.33, 0.96), Q4 for female: 0.37 (0.23, 0.58)]. People <60 years old had a lower risk of all-cause mortality with gynoid fat mass in Q3 than those ≥60 years old [<60 years: 0.50 (0.27, 0.91), ≥60 years: 0.65 (0.45, 0.95)]. Among subjects without hypertension, the group with the largest android fat mass had the lowest risk of CVD mortality, and the group with the largest gynoid fat mass had the lowest risk of all-cause mortality [Android fat mass: 0.36 (0.16, 0.81), gynoid fat mass: 0.57 (0.39, 0.85)].

**Conclusion:**

Moderate android fat mass and gynoid fat mass (Q3) had the most protective effect. Smokers and drinkers need to control their body fat. Being too thin is harmful to people with diabetes. Increased gynoid fat mass is a protective factor for all-cause mortality in older adults and females. Young people's gynoid fat mass is more protective in the moderate range than older people's. If no high blood pressure exists, people with more android and gynoid fat mass have a lower risk of CVD or all-cause mortality.

## Introduction

Obesity is associated with dyslipidemia, impaired glucose tolerance, and arterial hypertension, making it a significant risk factor for cardiovascular and metabolic diseases (1). Central obesity is associated with a higher risk of CVD (2). Even when there are no signs or symptoms of widespread obesity, excessive central adiposity is linked to metabolic abnormalities that raise the risk of CVD and diabetes (3). The association between abdominal fat accumulation and CVD appears to be more significant regarding the location of fat accumulation. Among the various metrics used to measure abdominal fat, using more accurate site-specific body fat measurements may provide a better understanding of the role of abdominal fat accumulation in CVD (4), and may also be used to predict CVD mortality.

The predictive power of various local obesity indicators for mortality has been validated (5, 6). Among them, men tend to accumulate adipose tissue in the abdominal region (Android fat mass distribution pattern) (7, 8). Android fat stores are mainly around the trunk/abdominal area (called “central” fat stores) or the upper body. Due to this distribution, those who exhibit excessive android fat patterns will portray an “apple” shaped appearance with a waist much larger than the hips. Android fat mass measures abdominal fat, including subcutaneous and visceral fat, and it has been confirmed to be a significant predictor of adverse cardiovascular events (9). Android fat mass was also associated with rheumatic diseases, metabolic syndrome, and diabetes (10, 11), suggesting that android fat mass is associated with a higher level of all-cause mortality.

On the other hand, some people tend to accumulate adipose tissue in the femoral-hip region (Gynoid fat mass distribution pattern) (7, 8) and are characterized by the accumulation of excess fat around the buttocks, hips, and thigh area. Due to this distribution, people may have a “pear” shaped appearance where the hips and buttocks fat is much larger than the waist. This type of distribution is typical in women (the term “gynoid” is associated with the female form). Gynecologic fat is stored “subcutaneously” under the skin and above the muscles. However, the results are controversial. Some studies have shown that gynoid fat mass, like android fat mass, was associated with increased cardiovascular risk (12–14) and various disease risks (15, 16). It may also raise the burden on heart failure patients (17). However, a study reported that, android fat mass distribution is a risk factor for CVD, but gynoid fat mass distribution pattern is not, according to Kyoung-Bok Min et al. (18). Some studies showed there was a potential protective effect of gynoid fat mass on CVD and mortality (15, 19–21).

There is few study using android fat mass and gynoid fat mass simultaneously to assess CVD and all-cause mortality, most previous studies focused on the risk of android fat mass and gynoid fat mass on cardiovascular events (12, 13). Our study will enrich the theory between android fat mass, gynoid fat mass and CVD mortality risk, and explore the association between android fat mass, gynoid fat mass and all-cause mortality. In addition, because of the standardized collection process and huge data volume of the NHANES database, it will be of great help to evaluate the relationship between android fat mass, gynoid fat mass and CVD and all-cause mortality. This study investigates the relationship between regional body fat [defined as android fat mass and gynoid fat mass in dual-energy x-ray absorptiometry (DXA)] and cardiovascular and all-cause mortality. We hypothesize that accurate measurement of site-specific body fat are the major predictors of cardiovascular and all-cause mortality.

## Methods

### Study subjects

The National Health and Nutrition Examination Survey (NHANES) is a population-based cohort study that collected data on adults and children's health and nutrition in the United States. The NHANES program began in the early 1960s as a series of surveys on different populations with various health and nutrition measures. The survey examines a nationally representative sample of approximately 5,000 people each year, randomly selected through a statistical process using U.S. Census information. NHANES defines baseline as the initial assessment of a participant's health status and risk factors at the beginning of the survey cycle. Follow-up refers to subsequent assessments conducted to track changes in health status and risk factors over time. NHANES is an ongoing, independent, nationally representative cross-sectional survey of non-institutionalized USA civilian populations conducted every 2 years. Interviews and physical tests are combined in this study, which is unique. The database includes demographic data, dietary, examination, laboratory, questionnaire, and limited access data (22).

**Figure 1:**
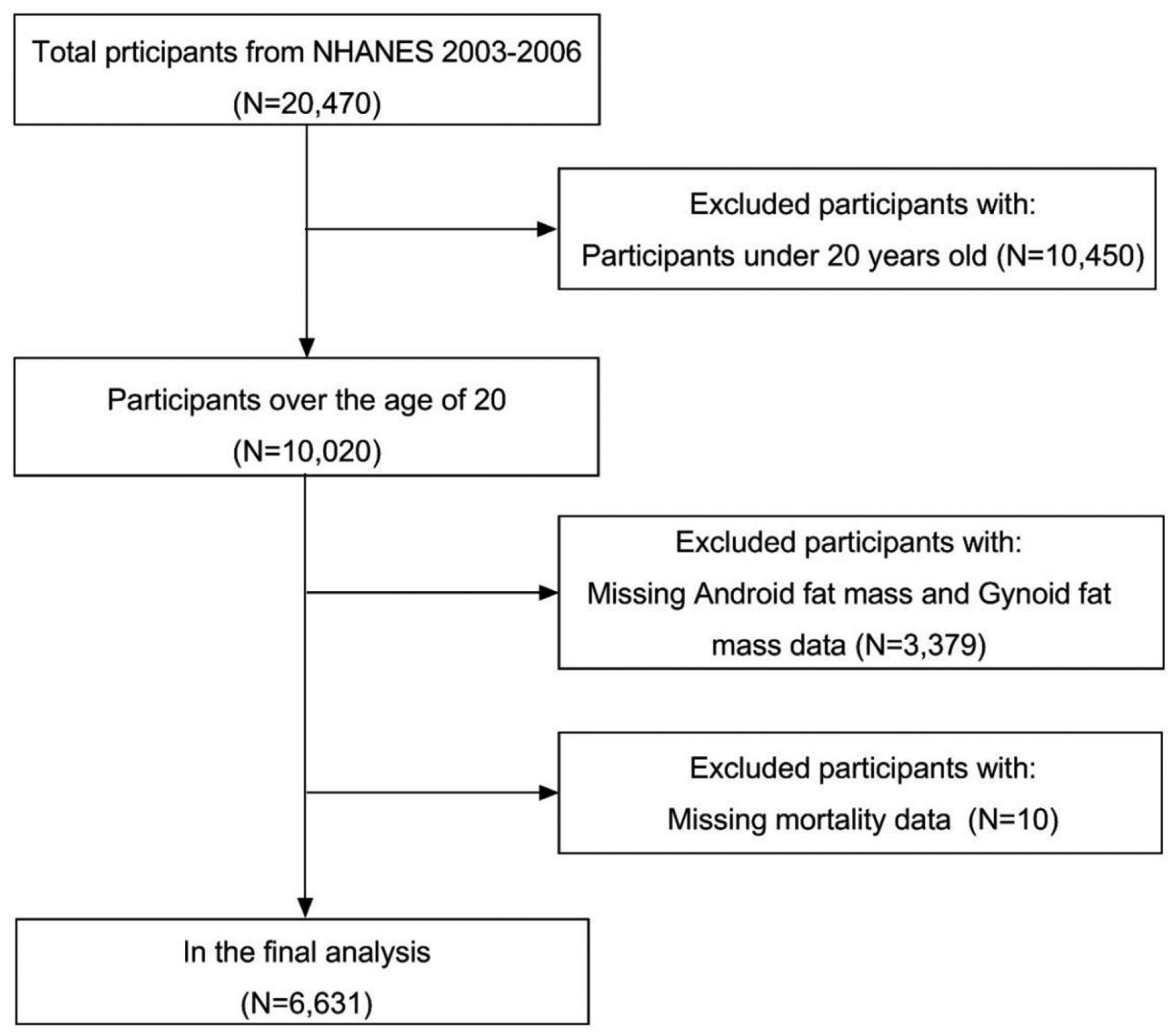
Study participant flowchart.

We analyzed 20,470 participants data in the NHANES database 2003-2007, excluding those under 20 years old, missing information on android fat mass, gynoid fat mass, mortality, and the final included 6,631 subjects (Figure 1).

### Exposure measurement

The International Society for Clinical Densitometry recommends Dual Energy x-ray Absorptiometry (DEXA) for measuring abdominal adiposity. DEXA's ability to accurately and precisely measure body fat mass in various body compartments has been well validated (23).

In NHANES, the android area is roughly the area around the waist between the mid-point of the lumbar spine and the top of the pelvis, and the gynoid area lies roughly between the head of the femur and mid-thigh. Whole-body DXA scans were obtained according to the manufacturer's recommended procedure on a QDR 4500A fan-beam densitometer. Certified radiology technologists administered the DXA examinations in the mobile examination center. All subjects were changed into paper gowns and were asked to remove all jewelry and other personal items that might interfere with the DXA examination. DXA exams were reviewed and analyzed by the UCSF Radiology Bone Densitometry Team using industry-standard techniques (24). All examinations were analyzed using Hologic Discovery software version 12.1 with default configuration, and the DXA instrument was calibrated as proposed by Schoeller et al. (25). The Hologic APEX software was used to define the android and gynoid regions in the scan analysis. Longitudinal monitoring was conducted through the daily spine phantom scans as required by the manufacturer, 3 times weekly whole-body slim-line phantom scans, and weekly air scans correct any scanner-related changes in participant data.

### Covariate assessment

Current study's covariates included age, gender, education, race/ethnicity, uric acid, total serum cholesterol, albumin, Vitamin C, folate, alcohol drinking, smoking status, history of diabetes, and hypertension. These data were collected from personal interviews, physical examinations, laboratory tests, nutritional assessments, and DNA repositories in the National Health and Nutrition Examination Survey (NHANES). Information on age, gender, race/ethnicity, education, drinking status and smoking status was self-reported by participants of NHANES via standardized questionnaires. Age was divided into three stages: <40, 40–60 and ≥60 years old. Race/ethnicity was defined as non-Hispanic white, non-Hispanic black, Mexican American and others. Education was classified as lower than high school, high school and higher than high school. Drinking status was categorized as none, moderate drinking (0.1–27.9 g/day for men and 0.1–13.9 g/day for women) and heavy drinking (more than 27.9 g/day for men and more than 13.9 g/day for women) (26). Average daily alcohol consumption derived from the alcohol use questionnaire was: [(quantity × frequency)/365.25]. Smoking status was classified as none smoker, a former smoker and a current smoker based on two inquiries: “Have you smoked at least 100 cigarettes in life and do you smoke cigarettes now?” (27). Laboratory data included uric acid, serum total cholesterol, albumin, vitamin C, and folate. Participants were invited to a Mobile Examination Center (MEC), where trained medical professionals collected blood samples. The blood sampling procedure involved drawing blood from a vein in the participant's arm using a needle and syringe. The blood sample was then sent to a laboratory for analysis. The results of the blood marker test were then added to the NHANES database along with other data collected during the exam. Beckman Synchron LX20 was used to measure serum uric acid levels by colorimetry; an enzymatic method was used to measure serum total cholesterol levels; albumin was measured by solid-phase fluorescent immunoassay; vitamin C was measured electrochemically by isocratic high-performance liquid chromatography at 650 mV, and serum folate was measured by Quantaphase II radiometry.

### Mortality data

Mortality status was ascertained from a linked mortality file prepared by the National Center for Health Statistics that contains a probabilistic match between NHANES and National Death Index (NDI) records; the follow-up time of this study was calculated from the examination date of NHANES 2003–2006 until the last known alive or censored date up to December 31, 2019. The mortality rate can accurately match each participant and be calculated as the number of deaths per unit of time (e.g., per year) among a specific population (e.g., by age group, race/ethnicity, or other demographic characteristics). Deaths from CVD were defined as deaths caused by cardiac diseases (codes I00–I09, I11, I13, I20–I51) or cerebrovascular diseases (codes I60–I69) (28). Meanwhile, we used sample weights to calculate estimates representing the U.S. civilian noninstitutionalized population or subpopulation. “PROC SURVEYREG” were used in computing descriptive and regression analyses.

### Statistical analysis

We quadrifid the participants according to android fat mass (Q1: <1,487.40 g, Q2: 1,487.40–2,208.79 g, Q3: 2,208.80–3,036.09 g, and Q4: ≥3,036.10 g) and gynoid fat mass (Q1: <3,116.20 g, Q2: 3,116.20–4,053.39 g, Q3: 4,053.40–5,235.79 g, and Q4: ≥5,235.80 g). The characteristics of the study population were expressed as frequencies and percentages, as well as averages and standard deviations. ANOVA for continuous variables and chi-square test for categorical variables were used to test hypotheses about the baseline features of the population with 4 levels of android fat mass and gynoid fat mass.

The hazard ratios and 95 percent confidence intervals between android fat mass, gynoid fat mass with CVD, and all-cause mortality were evaluated using a weighted Cox proportional risk regression model adjusted for various covariates. In model 1, we adjusted for age. In model 2, further adjusted gender, race/ethnicity, education level, alcohol status, and smoking status. In model 3, further added uric acid, total serum cholesterol, albumin, Vitamin C, and folate. Model 4 was a fully adjusted multivariate model using all the covariates from model 3 and a history of hypertension and diabetes.

In at least one model, age, gender, smoking status, drinking status, history of diabetes, and hypertension showed significant deviation from proportional risks (*P* < 0.05). Consequently, Cox regression analyses were stratified according to these factors, facilitating the computation of distinct hazard ratios within each stratum. SAS software version 9.4 (SAS Institute, Cary, North Carolina) was used to analyze the data, and a Two-sided *P* value <0.05 was considered statistically significant.

## Results

### Characteristics of the subjects

Android fat mass quantiles: Q1: <1,487.40 g; Q2: 1,487.40–2,208.79 g; Q3: 2,208.80–3,036.09 g; Q4: ≥3,036.10 g, and gynoid fat mass quartiles: Q1: <3,116.20 g; Q2: 3,116.20–4,053.39 g; Q3: 4,053.40–5,235.79 g; Q4: ≥5,235.80 g.

Throughout the 10.41 person-years of follow-up in this study (median follow-up 10.83 years; maximum follow-up 13.08 years), 882 deaths occurred, including 205 deaths from CVD. As shown in Table 1, the distribution of age, gender, race/ethnicity, education, smoking status, drinking status, total serum cholesterol, albumin, Vitamin C, folate, history of diabetes and hypertension for android fat mass and gynoid fat mass were statistically significant (*P *< 0.05) in the analysis of baseline characteristics. Participants with the highest android fat mass and gynoid fat mass were more likely to be 40–60 years old, female, non-Hispanic black, had a higher education level, never smoked, never drank alcohol, and had no history of diabetes and hypertension. The smallest android fat mass group had the lowest Uric acid, Serum total cholesterol, Folate and the highest Albumin and Vitamin C, while the smallest gynoid fat mass group had the lowest Uric acid, Serum total cholesterol, Folate, Vitamin C and the highest Albumin.

**Table 1:** Baseline demographic characteristics of the study population, according to android fat mass and gynoid fat mass.

Characteristic	Android fat mass				*P _trend_*	Gynoid fat mass				*P _trend_*
	Q1	Q2	Q3	Q4		Q1	Q2	Q3	Q4	
	<1,487.40	1,487.40–2,208.79	2,208.80–3,036.09	≥3,036.10		<3,116.20	3,116.20–4,053.39	4,053.40–5,235.79	≥5,235.80	
Total (*N*)	1,657	1,658	1,658	1,658		1,657	1,658	1,658	1,658	
**Age, years**					<.0001					<.0001
<40	914 (58.2)	607 (41.2)	449 (30.8)	449 (28.4)		686 (48.4)	610 (40.9)	531 (35.0)	592 (36.9)	
40–60	457 (32.2)	571 (40.8)	616 (47.6)	662 (50.2)		532 (37.3)	544 (42.2)	607 (44.4)	623 (45.6)	
≥60	286 (9.6)	480 (18.0)	593 (21.6)	547 (21.4)		439 (14.3)	504 (16.9)	520 (20.7)	443 (17.5)	
**Gender**					<.0001					<.0001
Men	879 (47.1)	899 (51.3)	871 (53.5)	779 (48.9)		1,340 (78.5)	1,026 (60.4)	711 (43.1)	351 (22.6)	
Women	778 (52.9)	759 (48.7)	787 (46.5)	879 (51.1)		317 (21.5)	632 (39.6)	947 (57.0)	1,307 (77.4)	
**Race/ethnicity**					<.0001					<.0001
Non-Hispanic white	854 (72.1)	758 (68.2)	798 (71.6)	912 (76.6)		708 (65.8)	853 (73.1)	908 (75.4)	853 (73.8)	
Non-Hispanic black	406 (11.4)	351 (11.2)	317 (9.7)	330 (10.0)		368 (11.4)	266 (7.6)	342 (10)	428 (13.3)	
Mexican American	249 (6.1)	397 (9.9)	422 (9.2)	333 (7.1)		420 (10.6)	396 (8.9)	298 (6.6)	287 (6.3)	
Other	148 (10.5)	152 (10.6)	121 (9.5)	83 (6.3)		161 (12.3)	143 (10.4)	110 (8)	90 (6.6)	
**Education**					<.0001					<.0001
<High school	159 (5.2)	237 (6.4)	269 (7.3)	167 (4.4)		299 (8.6)	232 (6.6)	170 (4.9)	131 (3.5)	
High school	603 (31.5)	625 (36.5)	654 (37.6)	682 (39.7)		635 (36.6)	624 (34.6)	616 (34.2)	689 (39.5)	
>High school	893 (63.4)	793 (57.1)	734 (55.0)	809 (55.9)		722 (54.8)	798 (58.8)	871 (60.9)	838 (57.1)	
**Smoking**					<.0001					<.0001
Never smoker	830 (50.5)	895 (52.6)	822 (48.8)	812 (48.2)		708 (42.8)	835 (50.5)	888 (51.1)	928 (54.5)	
Former smoker	298 (17.0)	369 (21.5)	472 (27.3)	481 (29.6)		395 (21.4)	420 (23.4)	424 (26.1)	382 (23.9)	
Current smoker	527 (32.5)	394 (25.9)	363 (23.9)	364 (22.3)		553 (35.8)	401 (26.1)	346 (22.8)	348 (21.6)	
**Alcohol**					<.0001					<.0001
Never drinker	1,087 (63.8)	1,131 (65.7)	1,187 (69.4)	1,279 (76.5)		1,052 (58.6)	1,117 (64.9)	1,189 (70.5)	1,326 (79.8)	
Moderate drinking	162 (10.5)	164 (11.1)	158 (9.7)	118 (7.6)		187 (13.0)	171 (11.0)	149 (9.0)	95 (6.3)	
Heavy drinking	360 (25.7)	310 (23.3)	271 (20.9)	229 (15.9)		365 (28.5)	321 (24.1)	281 (20.5)	203 (13.9)	
**Uric acid (umol/L)**	286.56 ± 71.52	312.74 ± 77.95	332.63 ± 80.84	348.45 ± 79.99	<.0001	318.74 ± 74.18	322.62 ± 82.99	320.17 ± 87.48	319.29 ± 78.71	<.0001
**Serum total cholesterol (mg/dl)**	187.73 ± 39.58	201.07 ± 41.15	207.13 ± 42.60	204.30 ± 44.67	<.0001	193.42 ± 42.71	201.59 ± 42.28	202.68 ± 41.77	202.63 ± 43.33	<.0001
**Albumin (g/dl)**	4.34 ± 0.35	4.27 ± 0.31	4.23 ± 0.32	4.13 ± 0.32	<.0001	4.35 ± 0.33	4.31 ± 0.32	4.23 ± 0.32	4.09 ± 0.3	<.0001
**Vitamin C (mg/dl)**	1.02 ± 0.52	0.97 ± 0.5	0.92 ± 0.47	0.83 ± 0.46	<.0001	0.9 ± 0.48	0.96 ± 0.5	0.98 ± 0.51	0.9 ± 0.47	<.0001
**Folate (ng/ml RBC)**	259.29 ± 109.46	273.87 ± 120.28	286.61 ± 128.58	297.81 ± 135.11	<.0001	258.43 ± 104.24	285.04 ± 122.5	285.78 ± 130.79	288.28 ± 135.99	<.0001
**History of diabetes**					<.0001					<.0001
Yes	69 (2.5)	123 (4.4)	184 (7.5)	242 (11.2)		146 (5.1)	159 (6)	150 (6.5)	163 (7.7)	
No	1,578 (96.9)	1,516 (94.9)	1,451 (91.4)	1,374 (86.4)		1,494 (94.2)	1,478 (92.8)	1,487 (92.7)	1,460 (90.2)	
Borderline	10 (0.6)	17 (0.7)	22 (1.2)	42 (2.4)		15 (0.7)	20 (1.2)	21 (0.8)	35 (2.2)	
**History of hypertension**					<.0001					<.0001
Yes	262 (13.5)	436 (22.0)	619 (32.5)	716 (40.3)		376 (18.8)	501 (26.2)	556 (29.4)	600 (32.2)	
No	1,383 (86.5)	1,207 (78.0)	1,029 (67.5)	933 (59.7)		1,262 (81.2)	1,146 (73.8)	1,091 (70.6)	1,053 (67.8)	

### Android fat mass, gynoid fat mass, and CVD, all-cause mortality

Table 2 shows the associations between android fat mass, gynoid fat mass, CVD, and all-cause mortality. In the fully adjusted model, the multivariable HRs (95% CIs) of all-cause mortality were 0.80 (0.61–1.04) in Q2, 0.62 (0.46–0.82) in Q3 and 0.64 (0.49–0.83) in Q4 for android fat mass (*P*_trend _= 0.0019), and 0.77 (0.61–0.97) in Q2, 0.60 (0.46–0.79) in Q3 and 0.61 (0.42–0.88) in Q4 for gynoid fat mass (*P*_trend _= 0.0134), respectively. The association between android fat mass and gynoid fat mass and CVD mortality was non-significant. We found that Q3 in android fat mass and gynoid fat mass had the smallest risk of all-cause mortality.

**Table 2:** Associations of android fat mass and gynoid fat mass with cardiovascular and all-cause mortality in U.S. adults aged at least 20 years.

	Android fat mass				*P _trend_*	Gynoid fat mass				*P _trend_*
	Q1	Q2	Q3	Q4		Q1	Q2	Q3	Q4	
	<1,487.40	1,487.40–2,208.79	2,208.80–3,036.09	≥3,036.10		<3,116.20	3,116.20–4,053.39	4,053.40–5,235.79	≥5,235.80	
Total (*N*)	1,656	1,658	1,658	1,659		1,657	1,658	1,658	1,658	
**CVD**										
Deaths, No. (%)	45 (1.3)	52 (1.4)	47 (1.5)	61 (2.4)	<.0001	75 (2.3)	59 (1.8)	31 (1.1)	40 (1.5)	<.0001
Deaths/ person-years	232/17,167	274/17,115	297/17,400	389/17,320		366/16,529	358/17,238	194/17,458	274/17,778	
Unadjusted	1 [Reference]	1.11 (0.79, 1.55)	1.13 (0.69, 1.86)	1.80 (1.14, 2.84)	0.0149	1 [Reference]	0.73 (0.55, 0.98)	0.45 (0.26, 0.77)	0.61 (0.37, 0.99)	0.0504
Model 1	1 [Reference]	0.68 (0.49, 0.95)	0.58 (0.35, 0.95)	0.93 (0.60, 1.45)	0.8018	1 [Reference]	0.62 (0.47, 0.83)	0.32 (0.18, 0.57)	0.48 (0.29, 0.78)	0.0086
Model 2	1 [Reference]	0.71 (0.49, 1.01)	0.57 (0.34, 0.94)	0.96 (0.58, 1.57)	0.7796	1 [Reference]	0.73 (0.54, 0.99)	0.44 (0.23, 0.85)	0.76 (0.41, 1.40)	0.3414
Model3	1 [Reference]	0.69 (0.47, 1.03)	0.49 (0.28, 0.87)	0.77 (0.45, 1.33)	0.6518	1 [Reference]	0.67 (0.48, 0.93)	0.39 (0.20, 0.76)	0.59 (0.31, 1.13)	0.1248
Model 4	1 [Reference]	0.64 (0.44, 0.93)	0.39 (0.22, 0.71)	0.58 (0.35, 0.98)	0.1577	1 [Reference]	0.60 (0.43, 0.82)	0.36 (0.18, 0.69)	0.53 (0.28, 0.99)	0.0719
**All-cause mortality**										
Deaths No. (%)	180 (6.1)	234 (7.8)	223 (8.2)	245 (9.7)	<.0001	267 (9.3)	241 (8.2)	197 (7.2)	177 (7.2)	<.0001
Deaths/person-years	1,060/17,167	1,422/17,115	1,442/17,400	1,536/17,320		1,513/16,529	1,518/17,238	1,222/17,458	1,207/17,778	
Unadjusted	1 [Reference]	1.28 (1.01, 1.63)	1.32 (1.04, 1.69)	1.57 (1.29, 1.92)	0.0002	1 [Reference]	0.84 (0.69, 1.02)	0.73 (0.59, 0.90)	0.72 (0.57, 0.92)	0.0246
Model 1	1 [Reference]	0.84 (0.67, 1.06)	0.74 (0.60, 0.91)	0.89 (0.74, 1.07)	0.4270	1 [Reference]	0.73 (0.60, 0.89)	0.54 (0.44, 0.68)	0.59 (0.45, 0.76)	0.0008
Model 2	1 [Reference]	0.86 (0.67, 1.11)	0.73 (0.58, 0.93)	0.91 (0.74, 1.11)	0.5306	1 [Reference]	0.83 (0.67, 1.03)	0.68 (0.53, 0.87)	0.78 (0.59, 1.04)	0.1126
Model3	1 [Reference]	0.84 (0.64, 1.10)	0.69 (0.52, 0.92)	0.75 (0.57, 0.97)	0.0290	1 [Reference]	0.82 (0.65, 1.03)	0.64 (0.49, 0.83)	0.65 (0.46, 0.92)	0.0193
Model 4	1 [Reference]	0.80 (0.61, 1.04)	0.62 (0.46, 0.82)	0.64 (0.49, 0.83)	0.0019	1 [Reference]	0.77 (0.61, 0.97)	0.60 (0.46, 0.79)	0.61 (0.42, 0.88)	0.0134

Values are n or weighted hazard ratio (95% confidence interval). Model 1: adjusted for age-. Model 2: model 1 + sex, race/ethnicity, education level, alcohol, and smoking. Model 3: model 2 + uric acid, serum total cholesterol, albumin, vitamin C, folate (RBC). Model 4: model 3 + history of hypertension, history of diabetes.

**Figure 2:**
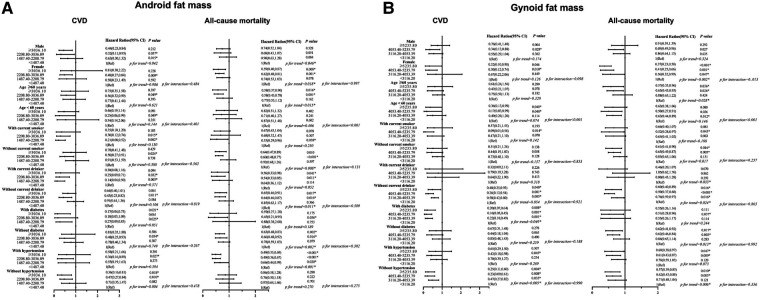
(**A,B**) Associations of android fat mass and gynoid fat mass with CVD and all-cause mortality Among different groups. Hazard ratios were adjusted for age, sex, race/ethnicity, education, alcohol, smoking, uric acid, serum total cholesterol, albumin, vitamin C, folate (RBC). History of Hypertension and history of diabetes. CVD, cardiovascular disease.

After that, we did a stratified analysis based on age, gender, smoking, alcohol, diabetes history, and hypertension history in Figure 2. When examining the relationship between android fat mass and CVD mortality, current smokers and current drinkers had the lowest CVD risk in Q2 [smoking: 0.21 (0.08, 0.52), drinking: 0.14 (0.04, 0.50)]. In diabetic patients, compared with Q1, other groups with increased android fat mass can significantly reduce the risk of CVD [Q4: 0.17 (0.04, 0.75), Q3: 0.18 (0.03, 1.09), Q2: 0.27 (0.09, 0.83)]. In ≥60 years old and female, the greater the gynoid fat mass, the smaller the HR of all-cause mortality [Q4 for ≥60 years old: 0.57 (0.33, 0.96), Q4 for female: 0.37 (0.23, 0.58)]. People <60 years old had a lower risk of all-cause mortality with gynoid fat mass in Q3 than those ≥60 years old [<60 years: 0.50 (0.27, 0.91), ≥60 years: 0.65 (0.45, 0.95)]. Among those without hypertension, the group with the largest android fat mass had the lowest risk of CVD mortality, and the group with the largest gynoid fat mass had the lowest risk of all-cause mortality [android fat mass: 0.36 (0.16, 0.81), gynoid fat mass: 0.57 (0.39, 0.85)].

## Discussion

In this large cohort study of the nationally-representative US population, android fat mass and gynoid fat mass were associated with risks of CVD and all-cause mortality. For older women, gynoid fat mass is a protective factor for all-cause mortality, when gynoid fat mass falls within the Q2–Q3 range, it exerts a more substantial protective effect on non-elderly individuals. Smokers and drinkers need to control their android fat mass to reduce CVD mortality, and being too thin can increase the risk of CVD mortality in diabetics. The non-hypertensive population with greater android fat mass and gynoid fat mass has a lower risk of CVD and all-cause mortality.

Obesity is a public health issue in high-income countries (29). Previous studies have shown that the amount of fat in the population has increased over the previous two decades, with the increase in younger age groups and women requiring special attention (30, 31). Obesity is measured by an increase in the number of adipocytes and excess fat stored in adipocytes. Body fat is mainly distributed in two main areas of the body. Therefore, it is often divided into subcutaneous adipose tissue (SAT) and visceral adipose tissue (VAT) (32). The latest research on android and gynoid fat mass represents a novel and intriguing direction, as android fat distribution describes the distribution of body fat tissue, primarily around the trunk and upper body, in regions such as the abdomen, chest, shoulders, and back of the neck. This pattern may result in an “apple” body shape or central obesity, with visceral adipose tissue being predominant (33). Excess visceral tissue increases susceptibility to a variety of metabolic diseases, including impaired glucose and lipid metabolism, insulin resistance (34, 35), colon cancer (36), breast cancer (37), and prostate cancer (38), as well as more extended hospital, stays more infectious and non-infectious complications, and higher in-hospital mortality (39). Gynoid fat mass is body fat that grows around the buttocks, chest, and thighs (40) and is primarily used to give nutrients to the offspring. It is also known as “reproductive fat” because it contains long-chain polyunsaturated fatty acids (PUFA), which are necessary for fetal development (33). Android and gynoid fat mass tissues have different cellular properties, with the android type having larger adipose (hypertrophic) cells and the gynoid type having more adipocytes (hyperplasia) (41). These discrepancies could explain their differing roles in lowering or raising mortality.

Compared to those in the lowest group, individuals with the highest android fat mass had increased albumin and vitamin C, and reduced serum total cholesterol and folate. Previous research on albumin and adiposity is limited. However, one study found a negative relationship between blood glycated albumin and body fat (42), similar to our findings, but the mechanism is unknown. Plasma vitamin C concentrations have been shown to correlate inversely with body mass index (BMI), percent body fat, and waist circumference (43). A positive relationship between serum total cholesterol and body fat has been reported (44). Folic acid is involved in the body's fatty acid metabolism (45), and several previous studies have found lower serum folate concentrations in individuals with higher body fat (45–47), contrary to our findings. However, one study found a positive association between obesity and erythrocyte folate (48).

It is well known that too little and too much body fat are risk factor (49). Its protective U-shaped effect is similar to BMI. In our study with Q1 as a control, the risk of mortality was reduced in all other groups with increased fat mass, which shows the protective effect of body fat (50), the ability to store and supply energy, improve the efficiency of sugar utilization, conserve protein, protect and insulating impact, and promoting the absorption of fat-soluble vitamins have been widely demonstrated (51–53). Q3 had the lowest HR of the three groups. When fat mass increased, the risk of both CVD and all-cause mortality decreased, then increased, which is consistent with earlier research. Excess fat mass is linked to an increased risk of mortality, according to Anja M Sedlmeier (54), who found a J-shaped association between the two; notably, there are also some studies pointing to a monotonic positive association between adiposity and mortality, and a wealth of studies have now demonstrated associations of various shapes, with one review indicating that adiposity was linked to mortality in either a positive monotonic or J-shaped relationship (55). In addition, a similar study focused on the impact of muscle and body fat on mortality. The same database found that specific subgroups with low fat had the lowest risk of death (50). Regarding the physiological function of android and gynoid fat mass, android-obese individuals have higher hematocrit, red blood cell count, and blood viscosity than gynoid-obese individuals. They are, therefore, more prone to CVD (56). However, compared to android fat, the ratio of gynoid fat to total fat amount is negatively associated with risk factors for CVD (14). Furthermore, both android and gynoid fat are important risk factors for CVD in children, particularly boys (12). Although it appears that android fat is a risk factor for CVD, different groups have reached different findings about whether the effects of gynoid fat are favorable or negative. In light of our findings, one possible explanation for why increased android and gynoid fat confers a lower risk of cardiovascular mortality was that the value of Q1 in our subgroup was too low and represents a relatively undernourished group, implying that any increase in fat mass would result in some reduction in cardiovascular risk and all-cause mortality. However the risk was lowest in Q3.

Among a stratified analysis of android fat mass and CVD mortality, it was discovered that current smokers and current drinkers have the lowest CVD risk in Q2, indicating that smokers and drinkers need to control their android fat mass at a small level because smoking, excessive alcohol consumption, and obesity are all risk factors for CVD death (57–59), combining smoking or alcohol abuse with obesity significantly increases CVD mortality (60, 61). Android fat mass represents central obesity, and individuals with central obesity have a higher risk of CVD death than those with general obesity (62); the risk of CVD death in current smokers or drinkers increases as android fat mass increases. We also found that compared to Q1, other diabetic patients with greater android fat mass can significantly reduce the risk of CVD. On the one hand, previous research has demonstrated that android fat mass functions as a mediator in metabolic syndrome (63), which could explain why diabetic individuals with higher fat mass have a lower risk of CVD death. On the other hand, too little fat mass can harm people with diabetes. Body fat regulates insulin sensitivity and glucose metabolism (64). Insulin resistance, a critical factor in developing of type 2 diabetes, is often associated with excess fat accumulation in the body (65). However, having too little body fat can also lead to insulin resistance and impaired glucose metabolism, especially in people with type 1 diabetes (66, 67). In addition, low body fat levels can lead to other health problems, such as nutrient deficiencies, compromised immune function, and decreased bone density (68).

In stratified analysis, increased gynoid fat mass reduced the risk of all-cause mortality in women and the elderly. This may be due to the close relationship between gynoid fat mass and estrogen (69); estrogen has a significant effect on cardiovascular protection (70) and body metabolism (71), and the characteristic part of gynoid fat mass: buttocks fat deposition was thought to be associated with better lipid and blood glucose levels (72). In a systematic review, increases in hip and thigh circumference were associated with a reduced risk of death, with the effect being more pronounced in women (73). Gynoid fat mass-type fat accumulation was inversely associated with CVD and type 2 diabetes (74); our findings confirm that discrete fat depots exert health effects uniquely, and that modest increases in gynoid fat mass may be more beneficial to female health. Also, research has demonstrated that gynoid fat mass protects older adults (75, 76); the reason may be that gynoid fat mass reduces the cardiovascular risk (14), improves glucose metabolism (77), and is associated with reduced injury from falls (76, 78). Furthermore, we found that the protective effect of gynoid fat mass decreased in the Q3 group from non-elderly to elderly. This may be due to hormonal changes that redistribute body fat from the hips and thighs to the abdomen (79), increased total body fat and decreased muscle mass due to decreased muscle mass and physical activity in older adults (80), and these factors reduce the protective effect of appropriate gynoid fat mass.

Last but not least, among those without hypertension, those with large android fat mass and gynoid fat mass had a smaller risk of CVD and all-cause mortality. A previous study confirmed that systolic blood pressure was positively correlated with visceral robotic fat (81). Studies prove that abdominal (android) fat mass was associated with high blood pressure. This was thought to be because the fat cells in this area release hormones and other substances that can affect blood pressure regulation (82). Excess weight can also contribute to high blood pressure, and clinical trials have shown a reduction in CVD mortality after lowering blood pressure (83). Excess regional fat appeared to be a risk factor for CVD and all-cause mortality in hypertensive populations. Because the health damage caused by larger android fat mass and gynoid fat mass overrides the health protection, especially the accumulation of visceral fat, leading to insulin resistance, elevated blood sugar levels, high blood pressure and dyslipidemia, atherosclerosis, all of which are risk factors for CVD (84, 85). But our study may show that the overall protective effect of large android fat mass and gynoid fat mass outweighs the risk in the non-hypertensive population, however, it was essential to note that the relationship between body fat distribution and health outcomes is complex and may be influenced by other factors such as age, sex, and lifestyle habits.

## Strengths and limitations

Based on the US NHANES database with a large population sample and established assessment methodologies, this study combined the effects of android and gynoid fat mass on CVD and all-cause mortality. However, there are a few flaws things to be aware of: (1) The time frame needs to be longer, and the follow-up years need to be longer. The NHANES database only investigated android fat mass and gynoid fat mass simultaneously and continuously during 2003–2007, which needs more context for the current study; (2) The index is too simple, and a new idea has been proposed in recent years: the obesity paradox, which states that people who are overweight or obese live longer, but our study does not define obesity criteria. Only fat mass was measured rather than a ratio, and the collected data are only absolute values, making it impossible to analyze an individual's fatness appropriately. Other studies have examined indicators, including the fat mass index (kg/m^2^), relative fat mass (RFM), and fat/lean ratio. Others have begun to look at the influence of the android/gynoid ratio on mortality; (3) The results of stratified analysis for diabetes differ from those of previous research. If our study could be divided into type 1 and type 2 diabetes, the results might have been better.

## Conclusion

The effects of android and gynoid fat mass on CVD and all-cause mortality in an adult population were investigated in this study. It was found that moderate android fat mass and gynoid fat mass (Q3) had the most potent protective effect. Smokers and drinkers need to control their body fat. Being too thin is harmful to the health of people with diabetes. Increased gynoid fat mass is a protective factor for all-cause mortality in older adults and females. Young people's gynoid fat mass is more protective in the moderate range than older people's. If no high blood pressure exists, people with more android and gynoid fat mass have a lower risk of CVD or all-cause mortality.

## Data Availability

Publicly available datasets were analyzed in this study. This data can be found here: https://www.cdc.gov/nchs/nhanes/.
